# Management of female uro-genital fistulas: Framing certain guidelines

**DOI:** 10.4103/0974-7796.62913

**Published:** 2010

**Authors:** R. B. Singh, Satish Dalal, S. Nanda, N. M. Pavithran

**Affiliations:** Departments of Burns and Plastic Surgery, Hypospadias and VVFs Clinic, Pt. B.D. Sharma Postgraduate Institute of Medical Sciences (P.G.I.M.S.), Rohtak-124 001, Haryana, India; 1 Department of General Surgery, Pt. B.D. Sharma Postgraduate Institute of Medical Sciences (P.G.I.M.S.), Rohtak-124 001, Haryana, India; 2 Department of Obstetrics and Gynecology, Pt. B.D. Sharma Postgraduate Institute of Medical Sciences (P.G.I.M.S.), Rohtak-124 001, Haryana, India

**Keywords:** Risky zone, safe zone, uro-genital fistulas, ureteric re-implantation

## Abstract

**Background:**

The study was carried out to discuss the pathogenesis and management protocol of seven different varieties of female uro-genital fistulas (FUGFs).

**Patients and Methods:**

During 2000–2007, total of 15 FUGFs were operated, which belonged to seven different varieties requiring different routes and surgical procedures for their repair. Different fistulas with different pathophysiological factors required specific examinations and investigations preoperatively.

**Results:**

The results of the repaired FUGFs, following the general surgical principles, were acceptable with formation of only one residual fistula.

**Conclusions:**

Successful correction of FUGFs is a surgical challenge. Detailed history, through examination and planning, atraumatic tissue handling, routine use of the interposition or onlay reinforcement flaps and vigilant postoperative care were found the key factors in successful outcome of the repaired fistulas.

## INTRODUCTION

Female uro-genital fistulas formation is the worst complication that can still be seen in developing and developed countries. Lack of proper obstetric care and prolonged obstructed labour forms the main cause in developing countries, while radical hysterectomy and radiotherapy for malignancy is the leading cause in developed countries. This report will highlight seven different varieties of such fistulas focusing on their etiological factors and different surgical techniques used for their repairs. It will also help in framing necessary guidelines for prevention of such FUGFs.

## MATERIALS AND METHODS

During last six years, 15 cases of female urogenital fistulas (FUGFs) were hospitalised, investigated, pre-operatively planned and operated. Out of them seven had gross dissimilarities in their pathogenesis, morbid anatomy and surgical procedures performed upon them. All were subjected to detail history, mandatory examinations (bimanual, speculum, methylene blue swab test and cystoscopy); and necessary investigations (USG of pelvic organs, IVP, MRI, urine and vaginal swab culture and sensitivity, blood urea and serum creatinine). Age of the patients ranged from 35 years to 65 years. For surgery all the cases were positioned in supine with head side low (Trendlenberg's position) for transperitoneal - transvesical approach. Estrogen cream was not used preoperatively in any of the patient, but they were instructed to start pelvic floor exercises (Kegel exercises)[1] from the very first day of their examinations and continued till three months after repairs to gain maximum control on urination.

Antibiotics were started on the day before surgery and were continued till all the tubings were removed. All patients were put on semisolid diet and stool softeners to prevent straining during defecation. A roll gauge impregnated with 5% povidone-iodine mixed with K-Y jelly was packed into vaginal cavity a night before the surgery for: (a) decreasing the depth of pelvic cavity to facilitate surgery, (b) antiseptic purposes and (c) identification of surgical plane between bladder and vaginal cavity. Surgical principles of tissue handling and dissection in proper planes were strictly followed; bladder and vagina were repaired in two layers with one continuous and other interrupted sutures using 2-0 vicryl on round needle. Each repair was reinforced with an omental interposition flap and or a Martius onlay flap. After completion of repair, the vaginal cavity was washed and repacked with betadine roll-gauge that was removed after 24 hours; thereafter the vaginal douchings were continued with diluted betadine solution. Intraperitoneal pelvic drain, suprapubic catheter and Foley's cathether were inserted routinely and all these were properly positioned and adequately fixed to prevent their kinking and mechanical blockage. A vigilant postoperative care was ensured to all the patients especially to maintain high urine output (75–100 ml per hour) to prevent intraluminal blockage of catheters. Anticholinergics were not routinely used in postoperative period except in few cases where there was definite evidence of bladder spasm. In cases where bladder spasm was clinically evident, tab. Buscopan was used twice a day for one week.

**Figure 1:**
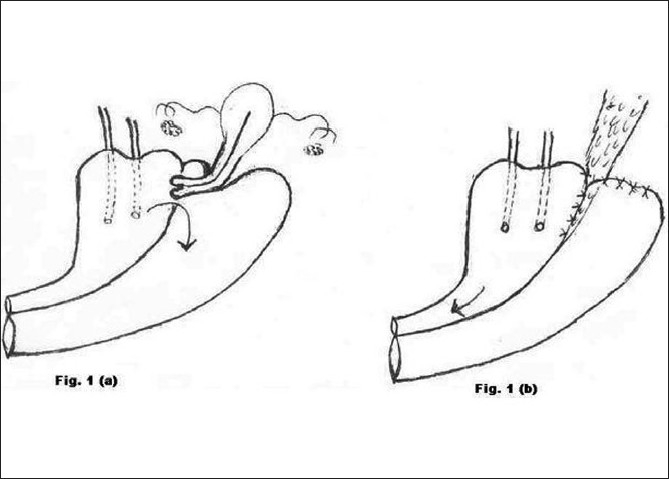
a) Preoperative morbid anatomy; b) Postoperative illustration

**Figure 2:**
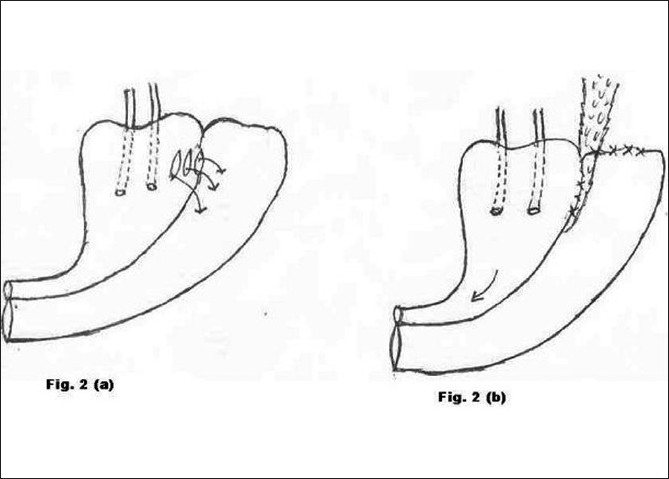
a) Preoperative morbid anatomy; b) Postoperative illustration

**Figure 3:**
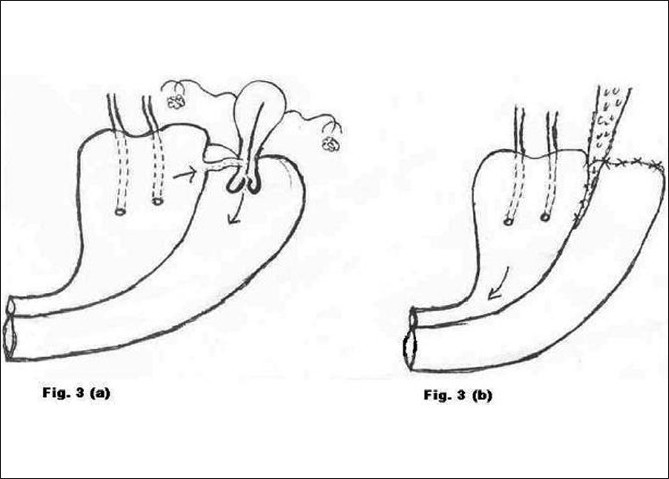
a) Preoperative morbid anatomy; b) Postoperative illustration

**Figure 4:**
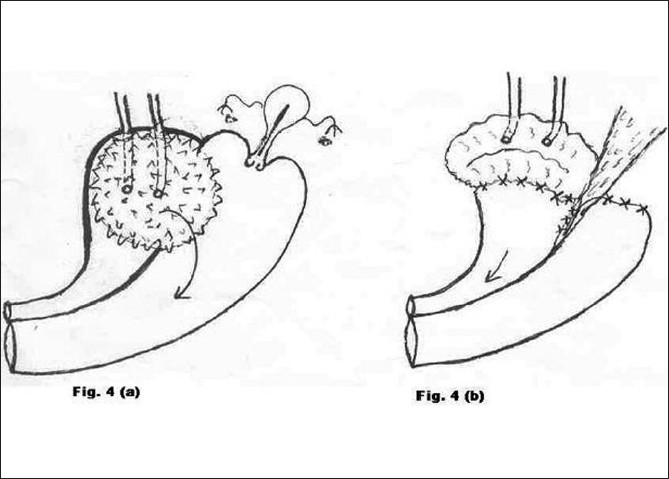
a) Preoperative morbid anatomy; b) Postoperative illustration

**Figure 5:**
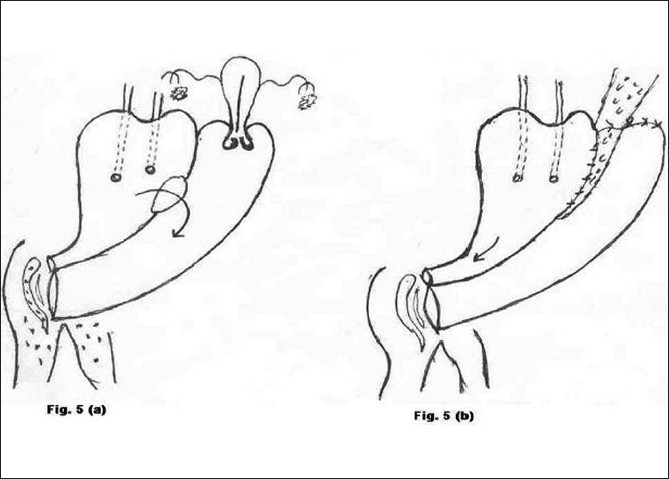
a) Preoperative morbid anatomy; b) Postoperative illustration

**Figure 6:**
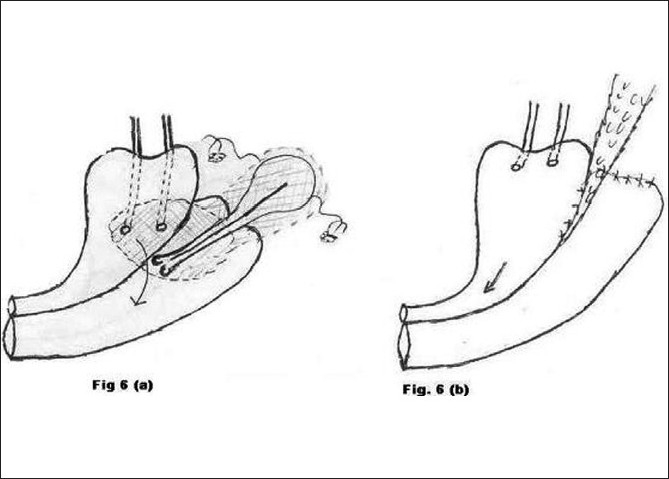
a) Preoperative morbid anatomy; b) Postoperative illustration

**Figure 7:**
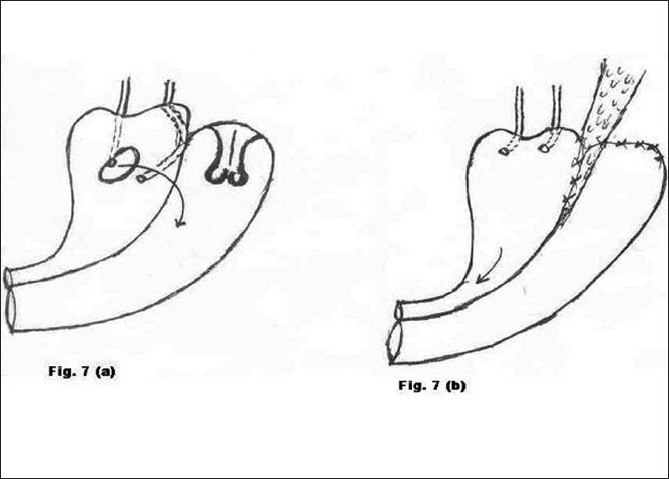
a) Preoperative morbid anatomy; b) Postoperative illustration

All the 15 cases were divided into seven categories depending on their common pathogenesis, presentations and surgical procedures performed [Table 1; Figure 1‐7].

**Table 1:** Showing seven different types of VVFs with different etiologies and surgical procedures performed upon them

Type	Etiology	No.of Cases	Morbid Anatomy and Surgical Procedure	Outcome
Type 1 [Figure 1]	Obstructed labour	3	Total hysterectomy with abdominal repair of VVF (Fig. 1a and 1b)	Successful
Type 2 [Figure 2]	Abdominal hysterectomy	4	Abdominal repair of VVF (Fig. 2a and 2b)	Successful
Type 3 [Figure 3]	Repeat LSCS	2	Abdominal repair of VVF (Fig. 3a and 3b)	Successful
Type 4 [Figure 4]	Neglected bladder stone	1	Total hysterectomy with Ileo-cystoplasty with ureteric reimplantation (Fig. 4a and 4b)	CISC
Type 5 [Figure 5]	Genito-urinary tuberculosis	1	Total hysterectomy with repair of bladder and vagina (Fig. 5a and 5b)	Recurrence
Type 6 [Figure 6]	Vaginal hysterectomy	2	Abdominal repair of bladder (Fig. 6a and 6b)	Successful
Type 7 [Figure 7]	Subtotal hysterectomy after LSCS	2	Excision of cervical stump and Abdominal repair of VVF (Fig. 7a and 7b)	Successful
Total		15	Successful outcome - 14, Recurrence - 1	

LSCS - Lower Segment Cesarean Section; VVF – Vesico-Vaginal Fistula; CISC – Clean Intermittent Self Catheterisation

## RESULTS

As evident from the Table 1, obstetrical trauma forms the main cause of vesico-vaginal fistulas (VVF) in our study i.e. three (obstructed labour) + two (Repeat LSCS) + two (subtotal hysterectomy to control bleeding); seven out of 15 cases were due to obstetric trauma. Second commonest cause was post-hysterectomy VVF, abdominal (4) and vaginal (2) cases. Other rare causes were big oxalate stone and genito-urinary tuberculosis in one case each. All these cases were having complex VVF either due to their size (mega VVF) or due to their locations and were operated upon by a combined team consisting of a plastic surgeon, general surgeon and a gynaecologist. Repair was done by abdominal route in all cases and omentum was used as interposition flap in all cases except one (case 4). Patients were evaluated two weekly initially for three months and three monthly later depending on presence of symptoms. Abstinence from sexual intercourse was advised from minimum of three months in sexually active females.

Out of 15 repaired FUGFs, 13 had successful surgical outcome and these 13 cases had full control on their acts of micturitions even without stress incontinence. Of the remaining two cases, one case with ileo-cystoplasty (case 4) had desire to pass urine after catheter removal but could only evacuate the bladder with clean intermittent self catheterisation (CISC). Her status of urine control could not be ascertained after discharge because she lost to follow-up. Another case with genital tuberculosis (case 5) had small, 5 mm residual fistula, which was closed successfully six months later.

## DISCUSSION

The incidence of FUGFs varies in different countries and is reported differently in various studies.[2] The condition is a socially debilitating problem with important medicolegal implications. In the developing countries, FUGFs following prolonged obstructed labour are more common because of low standard of antenatal and obstetric care.[3] These fistulas are associated with extensive pressure necrosis of bladder walls and urethra (mega fistula); diffuse peri-fistular fibrosis; high incidence of recurrence and failure rates due to their large size and presence of ischaemic tissues.[3] In contrast the postsurgical fistulas are result of more direct and localised trauma to otherwise healthy tissue, so having better results after repair.[4] Obstetric trauma remains the predominant cause in our series that gave rise to mega fistulas secondary to field injury effect which is comparable to previously reported series.[3] However, the majority of posthysterectomy fistulas in the present series were less than 2.5 cm size which is again comparable to other previously reported series.[3,4]

Various methods of fistula repair have been described in literature and include open transabdominal, transvaginal, laparoscopic, transurethral endoscopic and urinary diversion depending on the characteristics of the fistula.[4‐6] The best approach for complex fistulas is transabdominal using the O'Connors bivalve technique.[7] Patients with small bladders need augmentation cystoplasty in addition to the VVF repair. We had one such patient with neglected oxalate stone, leading to extensive scarring of bladder which was managed with ileo-cystoplastic augmentation of bladder with bilateral ureteric re-implantation. The success rate has varied between 75-95% with these techniques.[3‐7] Because of reasonable experience and expertise of our team, we could achieve success rate of 95% with formation of only one recurrent fistula out of 15 cases. Kegel's pelvic floor exercises were started preoperatively and continued postoperatively for a minimum period of three months to increase bladder capacity and urethral sphincter tone. During childbirth in prolonged obstructed labour, there is excessive stretching/contusions of pelvic floor muscles, bladder neck, internal and external urethral sphincters and anal canal sphincters. For having normal act of urination and to restore normal bladder capacity and urethral sphincter tone, it is mandatory to start these exercises.[1,2]

Inspite of the management being better defined and standardised over the last decade the surgical approach has always been an issue of contention. The fundamental principles of repair i.e. adequate exposure, tension free approximation of fistula edges, non-overlapping suture lines, multilayered closure of bladder and vagina at right angle to each other, good haemostasis and adequate postoperative bladder drainage can be achieved through both vaginal and abdominal route. However when fistula is complex, vaginal exposure of fistula is suboptimal which may compromise the repair or endanger the ureters. In these cases, a transabdominal approach should be preferred.[7] Nowadays all repairs are mandatory to be strengthened with routine use of reinforcement flaps, for which number of such flaps are described in literature with their multifactorial mechanism of action.[8,9] Omental flap in transperitoneal and Martius flap in transvaginal approach are two such reinforcement flaps, which are most versatile and vascular, and can be harvested with ease without producing any functional or cosmetic donor site deformities.[8,10]

In view of the observations made while dealing with different FUGFs along with review of some recent literature following comments are being made for the operating team.[11] All the members of operating team should be:

‐ Well conversant with normal and morbid anatomy of pelvis.
‐ Familiar with different surgical approaches and techniques.
‐ Aware of all relevant urological procedures on bladder, urethra and ureters.
‐ Expert in harvesting of need based reinforcement (interposition or onlay) flaps.
‐ Have an updated knowledge of preventing FUGFs under adverse circumstances like repeat LSCS, subtotal hysterectomy for control of bleeding, multiple large fibroids distorting pelvic anatomy and frozen pelvis due to sepsis/endometriosis etc.[12]

In the end remember that vast majority of fistulas are preventable. Prevention of such fistulas, include antenatal education, awareness of women in their reproduction periods, easily available transport facilities and widespread availability of obstetrical facilities even in remote rural areas.

## CONCLUSION

Surgical correction of FUGFs is still a great challenge and requires a team approach for better results. Such team must be well conversed with surgical anatomy and different procedures on various pelvic organs. We recommend transabdominal approach for complex fistulas, which also allows simultaneous correction of other morbidities and harvesting of different reinforcement flaps as per requirement is almost mandatory for favourable outcome.
